# Prevalence of occupational respiratory symptoms and associated factors among industry workers in Ethiopia: A systematic review and meta-analysis

**DOI:** 10.1371/journal.pone.0288238

**Published:** 2023-07-13

**Authors:** Chala Daba, Sisay Abebe Debela, Amanuel Atamo, Belay Desye, Mogesie Necho, Yonatal Mesfin Tefera, Fanos Yeshanew, Mesfin Gebrehiwot

**Affiliations:** 1 Department of Environmental Health, College of Medicine and Health Sciences, Wollo University, Dessie, Ethiopia; 2 Department of Public Health, College of Health Science, Salale University, Fitche, Ethiopia; 3 Department of Public Health, College of Medicine and Health Sciences, Adigrat University, Adigrat, Ethiopia; 4 Department of Psychiatry, College of Medicine and Health Sciences, Wollo University, Dessie, Ethiopia; 5 Adelaide Exposure Science and Health, School of Public Health, University of Adelaide, Adelaide, Australia; 6 School of Public Health, College of Medicine and Health Science, Wollo University, Dessie, Ethiopia; Wachemo University, ETHIOPIA

## Abstract

**Background:**

Occupational respiratory diseases are major global public health problems, particularly for industry workers. Several studies have investigated occupational respiratory symptoms in various parts of Ethiopia. The findings have been inconsistent and inconclusive, and there is no nationally representative data on the subject. Therefore, this study aimed to estimate the pooled prevalence and factors associated with occupational respiratory symptoms among industry workers in Ethiopia (2010–2022).

**Methods:**

Following the Preferred Reporting Items for Systematic Reviews and Meta-Analysis framework Guidelines, search was conducted on several international databases including PubMed, CINAHL, African Journals Online, Hinari, Global Health, and Google scholar. The extracted data was analyzed using STATA 14. Random effect model was used to estimate the effect size. Egger regression test and I^2^ statistics were used to determine potential publication bias and heterogeneity, respectively among the reviewed articles.

**Results:**

The meta-analysis included a total of 15 studies with 5,135 participants, revealing a pooled prevalence of 51.6% (95% CI: 43.6–59.6) for occupational respiratory symptoms among industry workers in Ethiopia. The absence of personal protective equipment (OR = 1.97, 95% CI: [1.17–3.32]), lack of occupational health and safety training (OR = 3.04, 95% CI: [2.36–3.93]), previous dust exposure (OR = 3.17, 95% CI: [2.3–4.37]), poor working environment (OR = 2.4, 95% CI: [1.7–3.2]), work experience greater than five years (OR = 4.04, 95% CI: [1.61–10.16]), smoking (OR = 6.91, 95% CI: [2.94–16.2]), and previous respiratory illness (OR = 4.25, 95% CI: [2.44–7.42]) were found to associate with the symptoms.

**Conclusions:**

The high prevalence of occupational respiratory symptoms among industry workers in Ethiopia underscores the urgent need for effective interventions. The provision of personal protective equipment and improvement of working environments by the government, industry owners, and other stakeholders are crucial in reducing occupational respiratory symptoms. Additionally, prioritizing occupational health and safety training for industry workers can help prevent and mitigate the impact of occupational respiratory diseases.

**Registration:**

This systematic review has been registered in the International Prospective Registry of Systematic Review (PROSPERO) with a specific registration number CRD42022383745.

## Introduction

Occupational respiratory diseases are significant public health concerns among industry workers worldwide. In 2010, the World Health Organisation (WHO) reported that 36 million industry workers suffered from occupational respiratory diseases and 4.2 million of them died due to exposure to occupational hazards [1]. Therefore, exposure to chemical and dust hazards at workplace remains the leading cause of public health problems [2] and increases the burden of occupational respiratory diseases [3]. According to the International Labour Organization (ILO), occupational respiratory diseases account for one-third (30%) of all registered work-related illnesses and 10–20% of deaths worldwide [4] and nearly similar is reported in different countries [5]. Driscoll et al (2005) also reported that occupational respiratory diseases due to exposure to respiratory hazards caused 386,000 deaths annually and resulted in nearly 6.6 million disability adjusted life years (DALYs) [6].

Indeed, the high prevalence of occupational respiratory diseases is reported in low and middle income countries due to poor working conditions, lack of training about occupational respiratory symptoms, and insufficient personal protective equipment [5]. In fact, according to Hamalainen (2017), of the 2.77 million of industry worker deaths, 17.1% death were caused by occupational respiratory diseases [7]. It is crucial to prioritize the health and safety of workers in the industry, as emphasized by the Sustainable Development Goals (SDGs) [8]. Many national and international organizations are focused on reducing the number of deaths and illnesses caused by occupational hazards. This is mainly implemented by the provision of training to industry workers and development of occupational health and safety policy [9].

In Ethiopia, despite all the efforts, occupational respiratory diseases remain major public health issues. In 2011, the World Health Organization (WHO) reported that the prevalence of occupational respiratory-related deaths in Ethiopia was 4%, which decreased to 3% in 2014 [10]. It is essential to determine the pooled prevalence of occupational respiratory symptoms and the factors that contribute to them in order to develop effective interventions for reducing occupational illnesses among industry workers. Although numerous studies have been conducted in different parts of Ethiopia, the findings have been inconsistent and inconclusive [11–22]. For example, the prevalence of occupational respiratory symptoms has been reported to vary from 32% among textile industry workers [12] to 69.8% among wood factory workers in Addis Ababa [13]. In other parts of Ethiopia, prevalence of occupational symptoms was found to be 58.3% in Addis Ababa [23], 47.8% in Amhara [14], and 50.8% in Oromia [24]. This review, therefore, aims to estimate the pooled prevalence of occupational respiratory symptoms and identify associated factors among industry workers in Ethiopia.

## Methods

### Search strategy

A systematic review and meta-analysis was carried out on both published and unpublished studies. Studies were searched through PubMed/Medline, CINAHL, African Journals Online, Hinari, Global Health, and Google scholar. The search was made using the search terms: "prevalence", "proportion", "magnitude", "incidence", "occupational respiratory symptoms", "respiratory symptoms", "industry", "factory", "small scale industry", "small scale enterprise", "workers", "small scale industry workers", "large scale industry", "large scale industry workers", "textile industry", "tannery industry", "flour industry", "construction factory", "Fuel station", "cement industry", "cotton ginning industry", "wood industry", "metal industry", "factors", "determinants", "predictors", "factors associated", "associated factors", "risk factors", "Ethiopia". All key terms were combined using Boolean operators “AND” or “OR” as appropriate and the search was done by two authors independently (CD and BD).

### Study selection and data extraction

This meta-analysis followed the Preferred Reporting Items for Systematic Reviews and Meta-Analysis (PRISMA) guidelines [25] (S1 Table). After searching relevant articles from selected electronic databases, they were exported to Endnote X20 and any duplicates were removed. Three authors (CD, BD, and MG) independently extracted all the required data from the included studies by using a standardized data extraction template on Microsoft Excel. The data extraction template consisted of various study details, such as the author’s name, region, publication year, study area, industry type, study setting, study design, sample size, response rate, and prevalence of occupational respiratory symptom. In case of any disagreements during data extraction, other authors (AA, YT, FY, and SAD) resolved it.

### Inclusion criteria

**Population:** This systematic review and meta-analysis includes studies conducted among industry workers in Ethiopia.

**Exposure**: Industry workers who experienced occupational respiratory symptoms.

**Comparison**: Individual workers who did not experience occupational respiratory symptoms.

**Outcome:** Studies assessed occupational respiratory symptoms as primary outcome.

**Study setting**: Institutional studies.

**Study design**: All observational studies (cohort, cross-sectional, and case-control).

**Publication**: Both published and unpublished studies.

**Country**: Studies conducted in Ethiopia.

**Language**: Studies published only in English language were included into the review.

### Exclusion criteria

Studies without full text, unidentified reports, editorials, letters to the editor, communications, case reports, case series, and qualitative studies were excluded from this study.

### Outcome measurement

The primary outcome of the study was to estimate the pooled prevalence of occupational respiratory symptoms among industry workers in Ethiopia, calculated by dividing the number of occupational respiratory symptoms by the total sample size and multiplying by 100. Additionally, the study aimed to identify the factors linked to occupational respiratory symptoms in the form of log odds ratio.

### Operational definition

Occupational respiratory symptoms: The development of one or more of the symptom(s), such as cough, phlegm, wheezing, shortness of breath, and chest tightness among workers [14, 17].

### Quality assessment

Following the removal of duplicate files using Endnote X20, four reviewers (CD, MN, MG, and SAD) screened the relevant articles for inclusion. The Joana Brigg Institute (JBI) critical appraisal checklist for prevalence studies was employed to evaluate the quality of each article [26] and those that scored more than 50% were included for analysis [27, 28] (S2 Table). Each study’s quality was assessed independently out of 100% by the five authors (CD, MN, MG, FY, and SAD). If any discrepancies arose during the quality assessment, the mean score was calculated from the results of all reviewers to resolve the differences.

### Statistical analysis

The data extracted using a Microsoft Excel spreadsheets were exported into STATA/ SE version-14 statistical software for analysis. The pooled estimate of occupational respiratory symptoms among industry workers in Ethiopia was computed using the random effects model of DerSimonian & Liard’s method at a significance level of p < 0.05 [29]. The level of heterogeneity among the included studies was statistically evaluated using the Higgs I^2^ test and funnel plot, with values greater than 75% considered high heterogeneity [30]. Additionally, publication bias was assessed using the Egger’s test method with a significance level of p < 0.05 [31].

## Results

### Study selection

**Fig 1:**
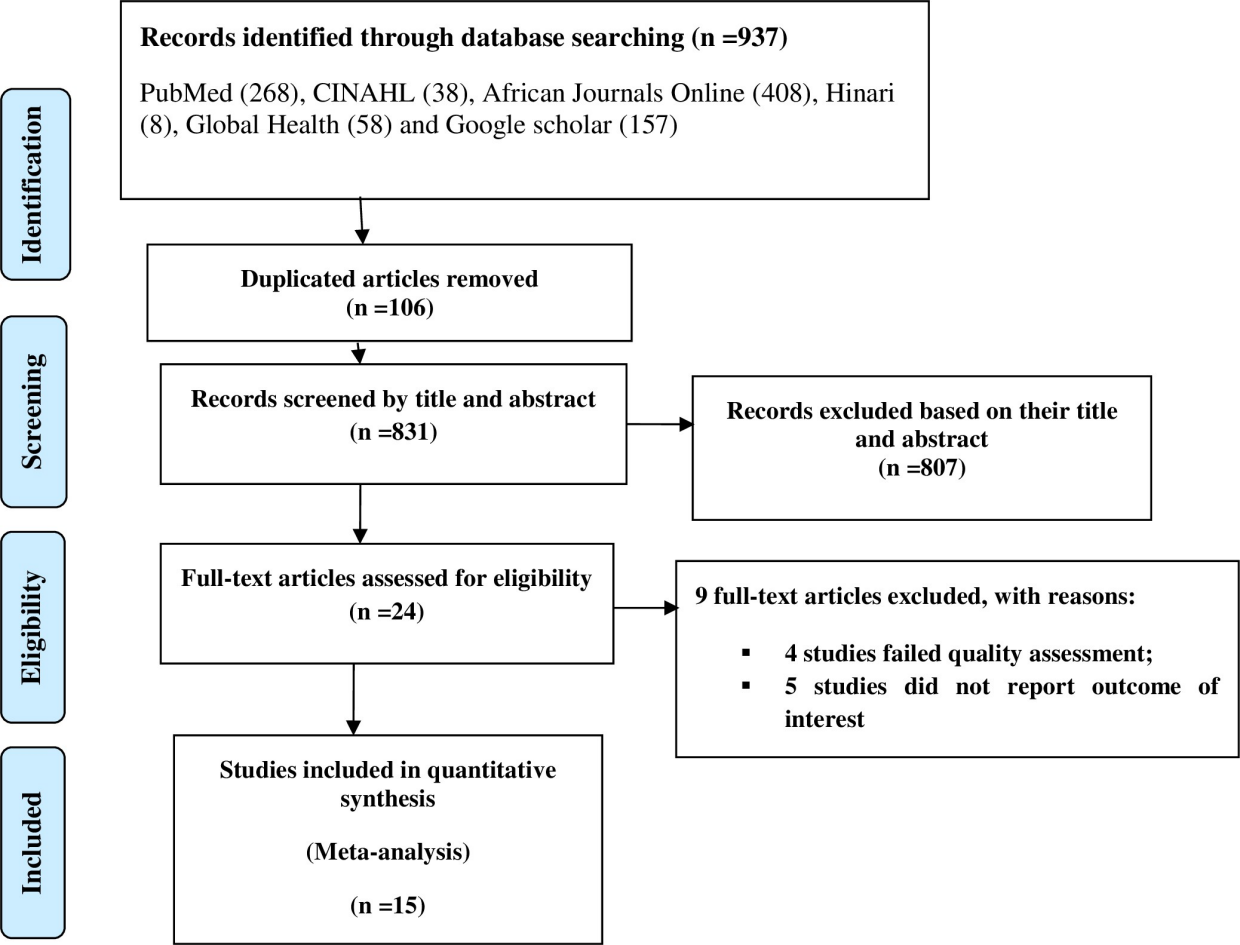
PRISMA flow diagram of the included studies for the systematic review and meta-analysis of occupational respiratory symptoms among industry workers in Ethiopia, 2023.

After conducting the electronic database search, a total of 937 articles were identified. A total of 106 duplicate articles were excluded, while 807 were excluded as they did not meet the inclusion criteria based on their titles and abstracts. Ultimately, 15 full-text articles were deemed eligible for the meta-analysis (Fig 1).

### Characteristics of the included studies

The final review consisted of 15 institutional-based cross-sectional studies that were published between 2010 and 2022 [11–24, 32], which included a total of 5,135 industry workers in Ethiopia. The study found that the highest prevalence of occupational respiratory symptoms (69.8%) was observed among wood industry workers in Addis Ababa [13], while the lowest prevalence (32%) was reported among textile industry workers in Addis Ababa [12]. In terms of location, five studies were conducted in the Ethiopian capital [11–13, 18, 23], six studies in the Amhara region [14, 16, 17, 19, 21, 32], three studies in the Oromia region [15, 22, 24], and one study in the Southern Nations nationalities and People’s Region (SNNPR) [20] (Table 1).

**Table 1:** Descriptive summary of fifteen studies included estimating the pooled prevalence of occupational respiratory symptoms and associated factors among industry workers in Ethiopia, 2023.

Authors	Year of publication	Region	Type of industry	Study design	Sample size	Response rate	Prevalence	Quality score
Alemu *et al* [12]	2010	Addis Ababa	Textile industry	Cross sectional	417	NA	32	75%
Derso *et a*l [16]	2021	Amhara	Cotton ginning factory	Cross sectional	166	100	68.6	87.5%
Abay *et al* [11]	2019	Addis Ababa	Fuel station	Cross sectional	394	89.8	48.7	75%
kifle *et al* [19]	2020	Amhara	Textile industry	Cross sectional	384	99.7	36.8	100%
Siyoum *et al* [22]	2014	Oromia	Cement industry	Cross sectional	535	100	66.2	87.5%
Alemseged *et al* [23]	2020	Addis Ababa	Flour factory	Cross sectional	424	97.8	58.3	100%
Awoke *et al* [13]	2021	Addis Ababa	Wood factory	Cross sectional	506	98	69.8	87.5%
Wami *et al* [14]	2018	Amhara	Texitle industry	Cross sectional	413	97.1	47.8	87.5%
Gizaw *et* al [17]	2015	Amhara	Cement industry	Cross sectional	404	100	62.9	87.5%
Dalju et al [15]	2019	Oromia	Tannery industry	Cross sectional	602	98	66.2	100%
Jabur et al [18]	2022	Addis Ababa	Wood factory	Cross sectional	232	99	65.7	87.5%
Lagiso et al [20]	2020	SNNPR	Flour factory	Cross sectional	406	96.7	56.6	100%
Mekonnen et al [21]	2021	Amhara	Flour factory	Cross sectional	560	100	63.9	87.5%
Tefera et al [32]	2020	Amhara	Tannery industry	Cross sectional	303	100	54	75%
Mekasha et al [24]	2018	Oromia	Cement industry	Cross sectional	309	97	50.8	87.5%

Note: NA- not available.

### Pooled prevalence of occupational respiratory symptoms

**Fig 2:**
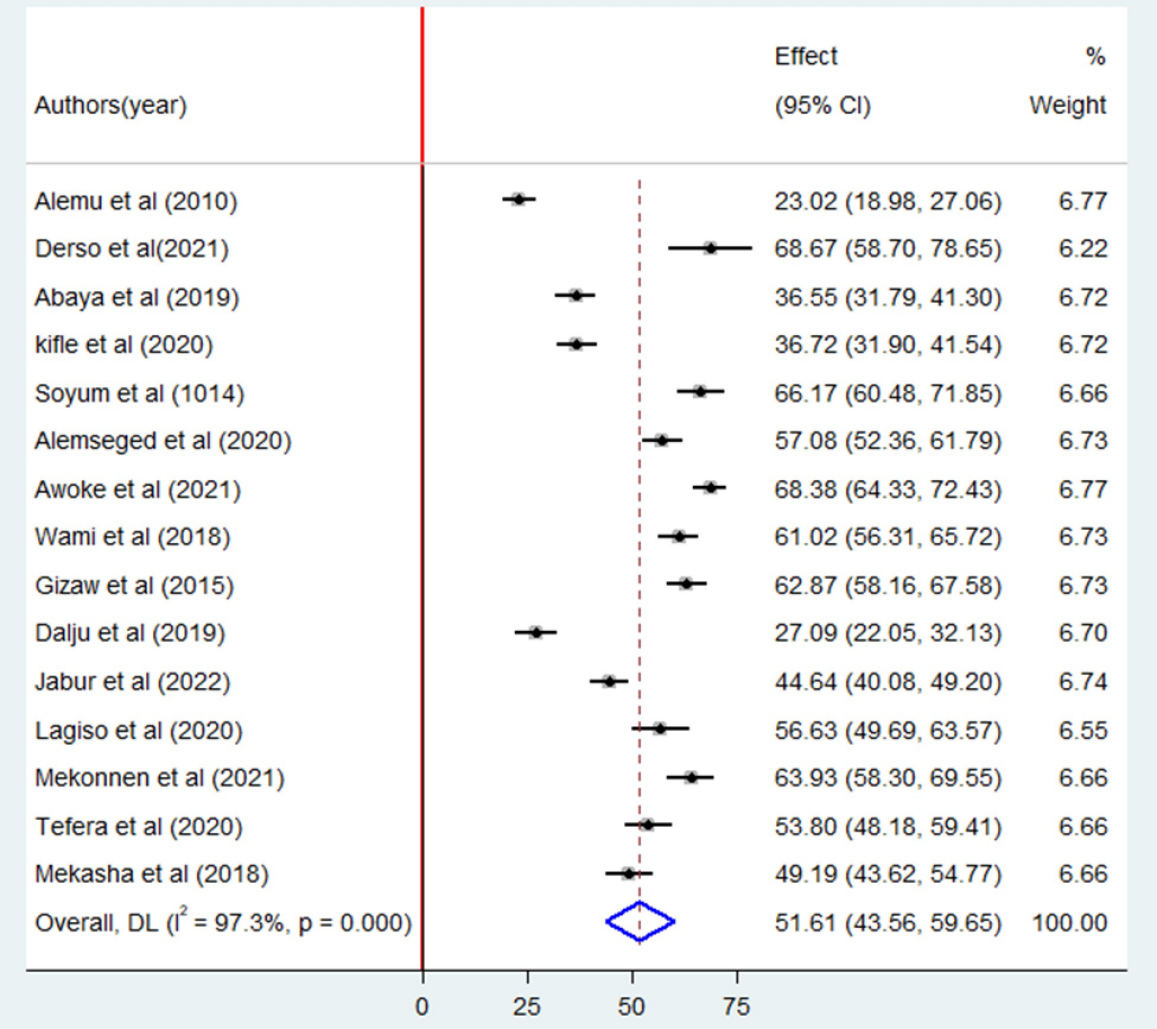
Forest plot of the pooled prevalence of occupational respiratory symptoms among industry workers in Ethiopia, 2023.

The results of 15 studies indicated that the pooled prevalence of occupational respiratory symptoms among industry workers was 51.6% (95% CI: 43.6–59.6). Due to high heterogeneity among the included research articles (I^2^ = 97.3%; P = 0.00), random effects model was employed to estimate the pooled prevalence (Fig 2).

### Publication bias

**Fig 3:**
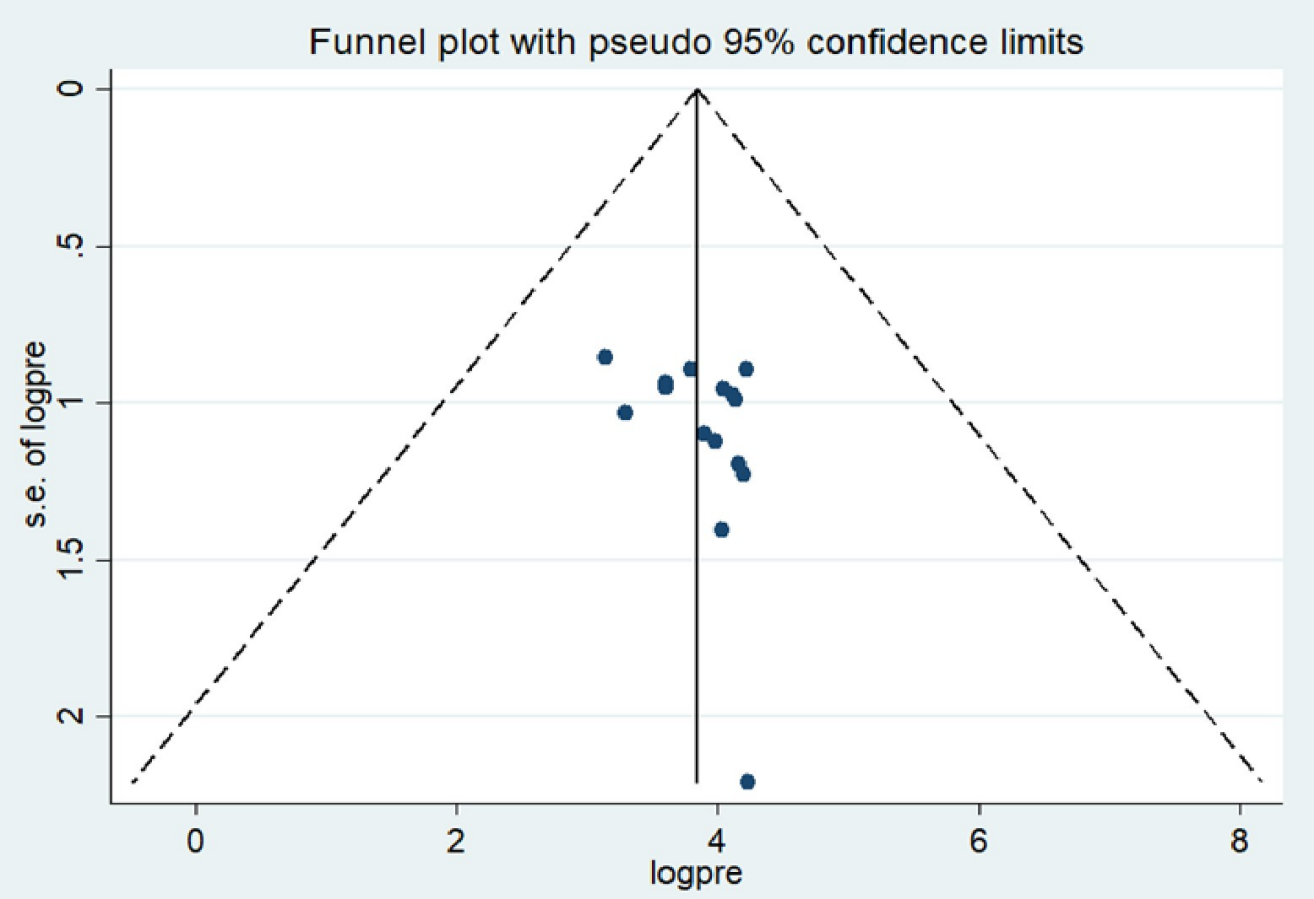
Funnel plot of the pooled prevalence of occupational respiratory symptoms among industry workers in Ethiopia, 2023.

Publication bias was subjectively evaluated by using funnel plot. The finding indicated that there is symmetrical distribution of the studies which suggests absence of publication bias (Fig 3). Moreover, the results from the egger’s test also showed that there was no significant publication bias (P = 0.187).

### Sensitivity analysis

**Fig 4:**
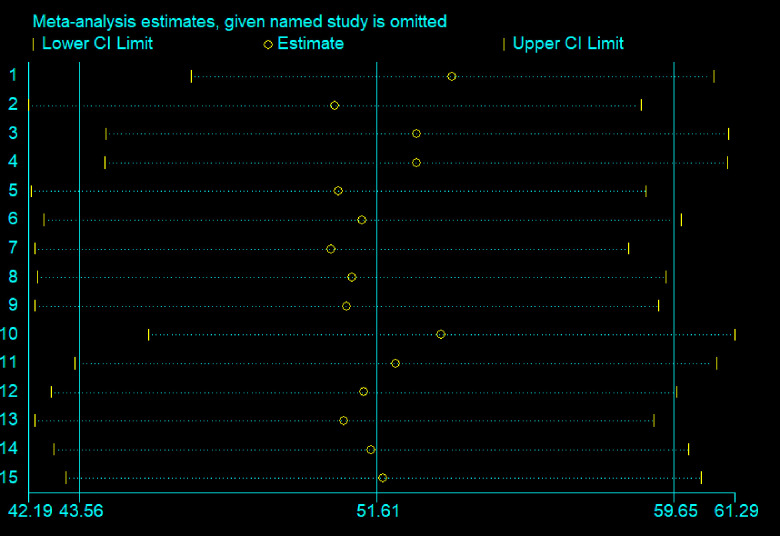
Sensitivity analysis for occupational respiratory symptoms, a systematic review and meta-analysis, Ethiopia, 2023.

Moreover, a sensitivity analysis was undertaken to ascertain a single study’s effect on the overall pooled estimate of occupational respiratory symptoms and the findings suggest no evidence of a single study effect on the overall pooled prevalence (Fig 4).

### Sub-group analysis

To identify the source of heterogeneity among the reports, sub-group analysis was undertaken based on region, type of industry, year of publication, and sample size. Accordingly, the highest pooled prevalence of occupational respiratory symptoms was reported among cement factory workers as compared to textile and other industry workers [56.01% (95% CI: 47.7–63.4)]. In addition, the highest prevalence of occupational respiratory symptoms was observed in the studies done in Amhara region as compared to studies done in Addis Ababa, SNNPR, and Oromia regions [57.59% (95% CI: 48.2–67.03)]. Regarding sample size, the highest prevalence was observed in studies with smallest sample size than others [56.36% (95% CI: 39.4–73.4)] (Table 2).

**Table 2:** Sub-group analysis of the pooled prevalence of occupational respiratory symptoms among industry workers in Ethiopia, 2023.

Sub-group	Number of studies	Sample size	Pooled prevalence (95% CI)	Heterogeneity	
				I^2^	p-value
**By type of industry**					
Textile industry	4	1513	36.9 (20.04–53.9)	98.1%	<0.001
Cement industry	3	979	59.4 (49.6–69.2)	90.2%	<0.001
Other industries*	8	2643	56.01 (47.7–63.4)	94.9%	<0.001
**By study year**					
2018 and before	5	1809	52.4 (35.1–69.7)	98.4%	<0.001
Post 2018	10	3326	51.2 (42.1–60.3)	96.7%	<0.001
**By region**					
Addis Ababa	5	2198	45.93(29.52–62.35)	98.6%	<0.001
Amhara	6	1867	57.59 (48.2–67.03)	94.3%	<0.001
Oromia	3	874	47.45 (24.9–70.01)	98.1%	<0.001
SNNPR	1	196	56.63 (49.69–63.57)	0.0%	-
**By sample size**					
Large	10	4011	49.32 (39.7–58.9)	97.6%	<0.001
Small	5	1124	56.36 (39.4–73.4)	97.3%	<0.001

*Other industry: fuel station, wood factory, cotton ginning, flour factory, and tannery industry

### Factors associated with occupational respiratory symptoms

In the systematic review and meta-analysis, there were nine factors frequently reported in the included studies. These factors were sex, training, educational status, work experience, previous dust exposure, personal protective, history respiratory symptoms, working environment, and smoking. Of these factors, previous dust exposure [11, 13, 23], smoking [17, 22], previous respiratory illness [17, 18], poor working environment [14, 15, 21], and work experience [13, 14, 17, 20, 21] were positively associated with increased respiratory symptoms.

A total of four studies [15, 16, 19, 23] were included to assess the association between personal protective equipment and occupational respiratory symptoms. The association was significant in three studies. The pooled meta-analysis revealed that the odds of occupational respiratory symptoms were two times higher among industry workers who did not use personal protective equipment (OR = 1.97; 95% CI: 1.17–3.32) (Table 3). The association between occupational respiratory symptoms and sex was examined based the results of six studies [14–18, 22]. Positive association was reported in three studies, while negative in the others. The result of random effects analysis showed non-significant association between occupational respiratory symptoms and sex (OR = 1.48; 95%CI: 0.91–2.40).

**Table 3:** The pooled effect size of factors associated with occupational respiratory symptoms among industry workers in Ethiopia, 2023.

Variables	Number of study participants	Number of study included	Odds ratio (95% CI)	Heterogeneity	
				I^2^	p-value
Absence PPE	1180	4	1.97 (1.17–3.32)	70.7%	0.017
Male	1919	6	1.47 (0.91–2.4)	81.8%	<0.001
No training	1745	5	3.04 (2.36–3.93)	0.00%	0.658
Previous dust exposure	1325	3	3.17 (2.3–4.37)	10.0%	0.239
Poor working environment	980	3	2.4 (1.7–3.2)	0.0%	0.969
Work experience greater than five years	1777	5	4.04 (1.61–10.16)	93.3	<0.001
Smoking	670	2	6.91 (2.94–16.2)	0.0%	0.810
Previous respiratory illness	856	2	4.25 (2.44–7.42)	10.3%	0.291
Having less than grade eight education	485	2	1.52 (0.97–2.38)	8.1%	0.297

**Fig 5:**
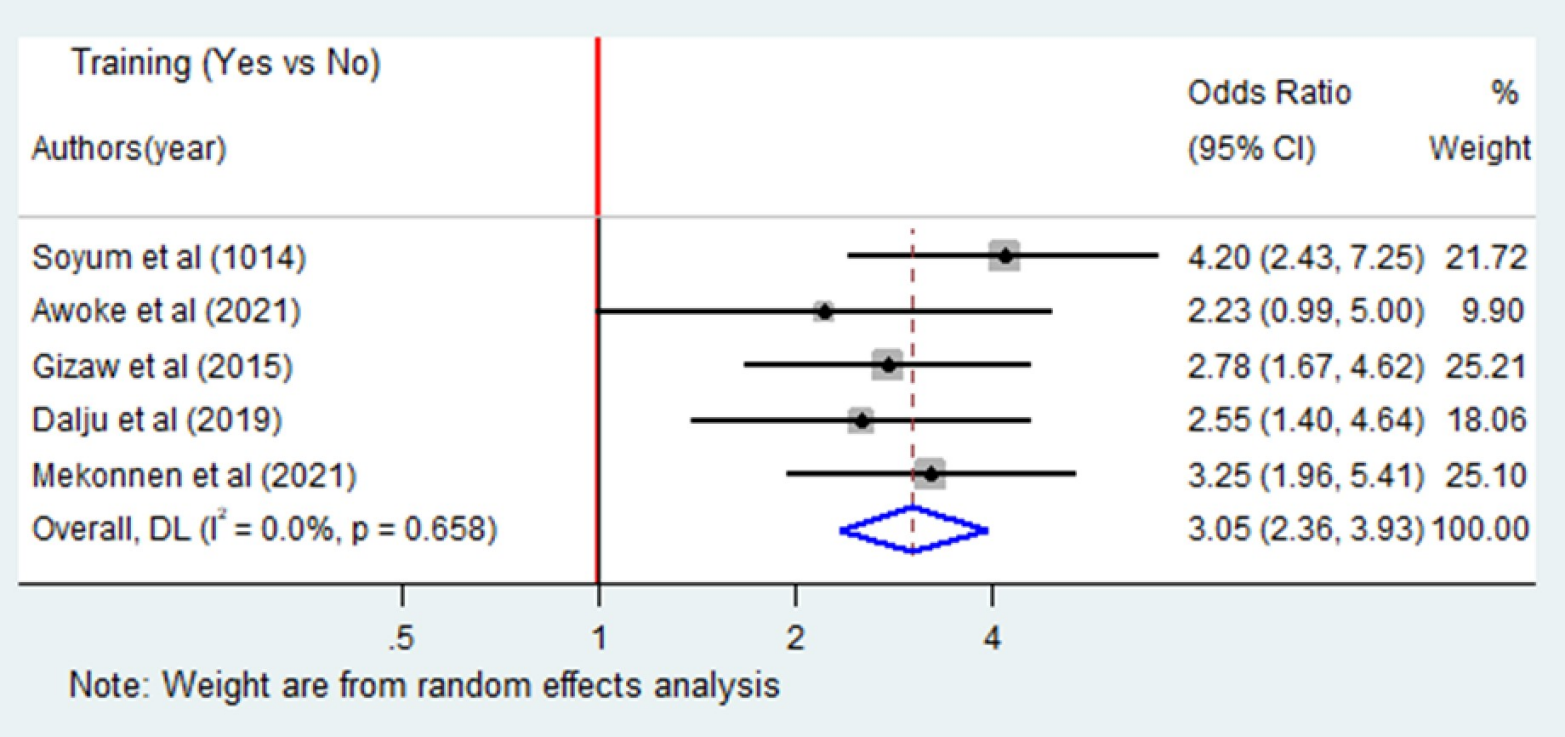
Forest plot of odds ratio for the association of training and occupational respiratory symptoms among industry workers in Ethiopia, 2023. Note: Weight are from random effects analysis.

Five articles [13, 15, 17, 21, 22] were included to identify the association between training and occupational respiratory symptoms. Four of the included studies had significant association. The results of random effects meta-analysis showed that the odds of occupational respiratory symptoms were 3.05 times higher among industry workers who did not receive occupational health and safety training (OR = 3.05; 95%CI: 2.36–3.93) (Fig 5). While the association between occupational respiratory symptoms and prior respiratory illnesses was examined based the results of two studies [17, 18], both indicated significant association. The pooled meta-analysis showed that the odds of occupational respiratory symptoms were four times higher among industry workers who had prior respiratory symptoms than others (OR = 4.25; 95% CI: 2.44–7.42).

**Fig 6:**
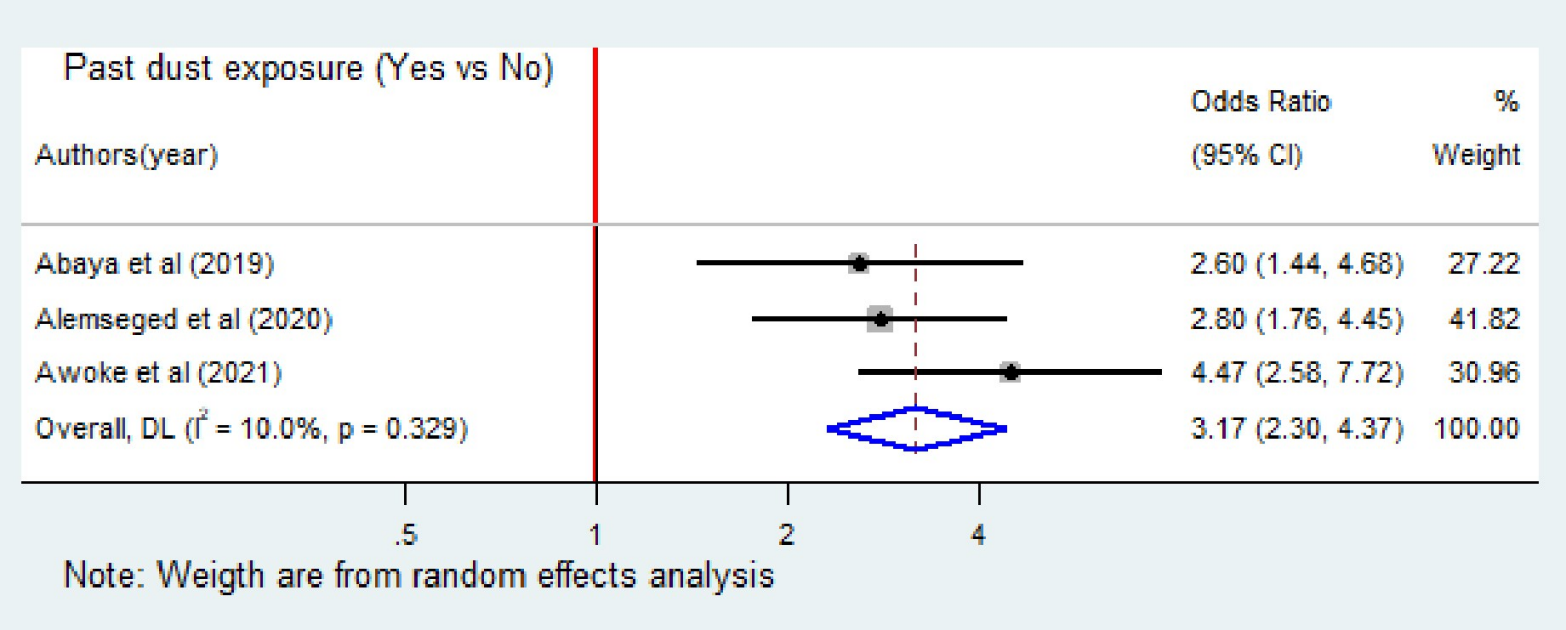
Forest plot of odds ratio for the association of past dust exposure and occupational respiratory symptoms among industry workers in Ethiopia, 2023. Note: Weight are from random effects analysis.

Similarly, a total of three studies [11, 13, 23] were included to assess the effect of previous dust exposure on occupational respiratory symptoms. In all the three studies, the association was found significant. Accordingly, industry workers who had previous dust exposure were 3.17 times more likely to develop occupational respiratory symptoms (OR = 3.17; 95% CI: 2.3–4.37) (Fig 6).

**Fig 7:**
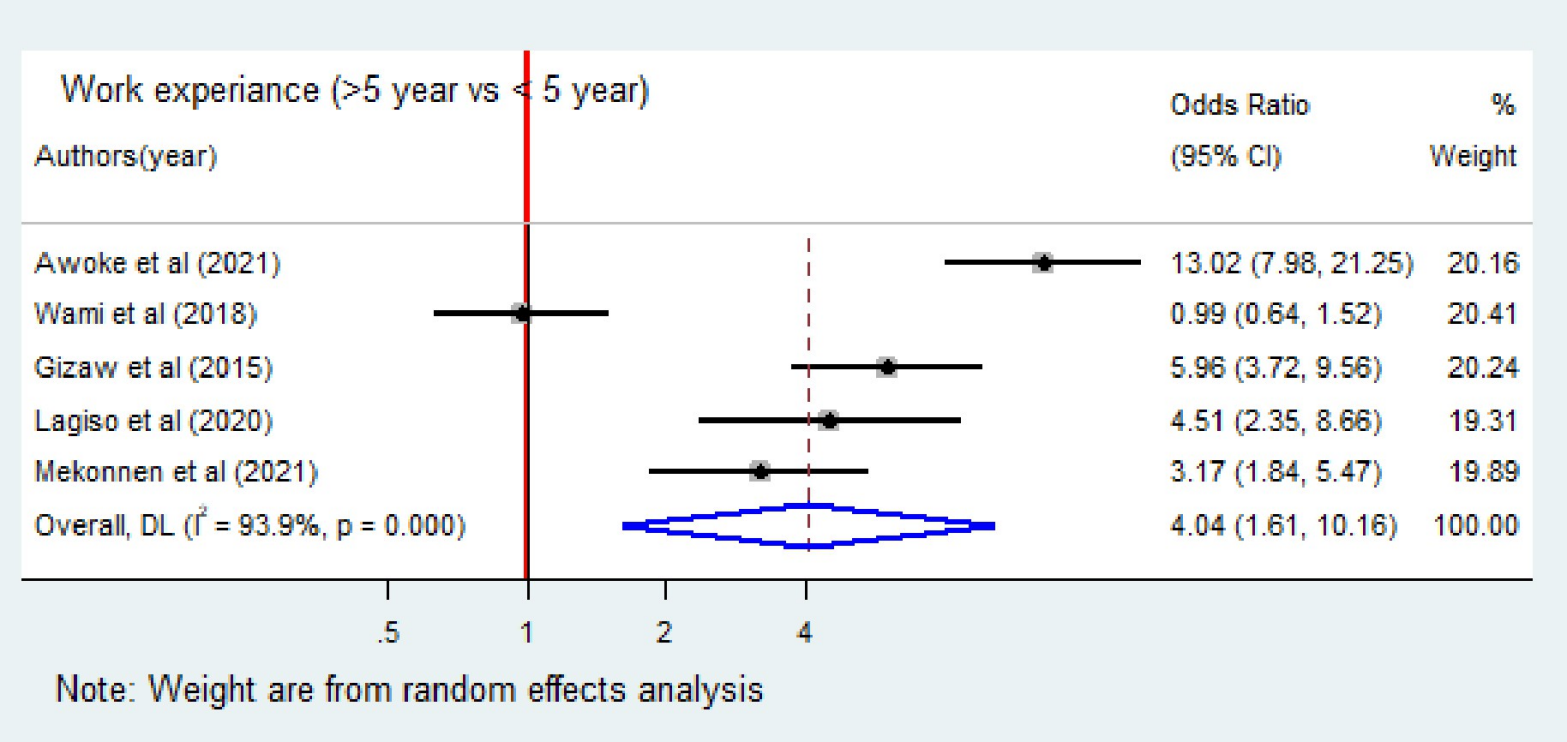
Forest plot of odds ratio for the association of working experience and occupational respiratory symptoms among industry workers in Ethiopia, 2023. Note: Weight are from random effects analysis.

The association between occupational respiratory symptoms and work experience was examined based the results of fives studies [13, 14, 17, 20, 21]. Positive association was reported in four studies, while negative in the other. The pooled meta-analysis showed that the odds of occupational respiratory symptoms were four times higher among industry workers who had more than five years of work experience than these who had less than five years of experience (OR = 4.04; 95% CI: 1.61–10.16) (Fig 7). Once more, two articles [17, 22] were included to identify the association between occupational respiratory symptoms and smoking. Based on the results, the odds of occupational respiratory symptoms were seven times higher among smokers than non-smokers (OR = 6.91; 95% CI: 2.94–16.2) (Table 3).

Additionally, three studies [14, 15, 21] were included to assess the association between working environment and occupational respiratory symptoms. All the three studies indicated significant association. The pooled meta-analysis also indicated that these working in poor environment were 2.3 times more likely to experience occupational respiratory symptoms (OR = 2.3; 95% CI: 1.7–3.2) than others. Although the association between occupational respiratory symptoms and level of education was examined based the results of two studies [17, 20], non-significant association (OR = 1.52, 95% CI: 0.97–2.38) was noted.

## Discussion

Occupational exposure to respiratory hazards has continued to be a global burden of occupational respiratory diseases. To identify the possible factors associated with the problem, various studies have been conducted. However, the results are inconsistent and inconclusive, which could complicate the design and implementation of effective intervention activities. Therefore, this systematic review and meta-analysis was conducted to estimate the pooled prevalence of occupational respiratory diseases among industry workers in Ethiopia. The pooled prevalence of occupational respiratory symptoms was found to be 51.6% (95% CI: 43.6–59.6). This figure is greater than these reported among industry workers in the United Kingdom (22%) [33], Eastern Nepal (21.1%) [34], Hong Kong (27.2%) [35], and Bangladesh (34%) [36]. Types of the industry and lack of personal protective equipment provision to industry workers might explain the discrepancies. The other possible reasons might be differences in the workers educational level. Community education, including that of industry workers in Ethiopia, is limited to lower classes. This is likely to reduce the workers’ compliance to occupational respiratory disease prevention measures and then increase the burden of the problem in our study. On the other hand, the pooled prevalence is consistent with studies conducted among woodworkers in Cameroon (51%) [37], Iran (53%) [38], and France (56.6%) [39].

The odds of occupational respiratory symptoms were three times higher among industry workers who did not receive occupational health and safety training than those who did receive the training. This finding is in line with other studies in Norway [40] and Egypt [41]. Industry workers without training could lack the required knowledge and skill to protect themselves from health effects associated with occupational respiratory hazards.

The pooled odds of experiencing occupational respiratory diseases among industry workers who had greater than five years of work experience were higher than those who had less than five years work experience. These finding is also in agreement with studies conducted in Ethiopia [42], Italy [43], Gujarat [44], Addis Ababa [45], Australia [46], India [47], Myanmar (Southeast Asia) [48], and Republic of Macedonia [49]. Workers exposed to occupational hazards for a long period of time in poor working environments could have an increased risk of developing occupational respiratory symptoms. Indeed, long-term exposure to chemicals and dusts could also lead to higher accumulation of dust in the respiratory tracts and then cause respiratory diseases [50]. The workers who had previous dust exposure were also three times more likely to experience occupational respiratory diseases than others. Our finding is supported by a study done in Tanzania among the sunflower oil industry workers [51].

Similarly, the pooled odds of occupational respiratory symptoms were two times higher among industry workers who did not use personal protective equipment (PPE) than their counterparts. This finding is in congruent with studies done in Hawassa [52], India [53], Pakistan [54], and Nigeria [55]. Besides the lack of PPE, other related factors, such as misuse, lack of strict follow-up and regular supervision as well as lack of occupational health and safety training could justify. On the other hand, other studies conducted in Ethiopia [45], Tanzania [40], and Nigeria [56] reported no difference in occupational respiratory symptoms between personal protective equipment users and non-users. This discrepancy might be linked to the quality of personal protective equipment used and other measures in place to ensure PPE use compliance.

The workers who had previous respiratory diseases were four times more likely to experience occupational respiratory symptoms than their counterparts. It is in line with a study conducted in Addis Ababa (46). In this meta-analysis, smoking was also positively associated with occupational respiratory symptoms. The workers who had smoking habit were six times more likely to develop occupational respiratory symptoms than others. This finding is similar with studies conducted in Iran [57], India [58], and Italy [59].

The odds of occupational respiratory symptoms were almost two times higher among industry workers who work in poor working conditions. This finding is supported by other studies conducted in the United States [60] and China [61]. Poor working environment could increase workers’ exposure to contaminants and respiratory hazards. For example, occupational exposure to fiber dust and chemical agents in poor working conditions may result in occupational respiratory diseases [62, 63]. Similarly, the odds of occupational respiratory symptoms were almost three times higher among those who had previous dust exposure than their counterparts. This finding is consistent with the study done in Tanzania among sunflower oil industry workers, which indicated that previously dust-exposed workers were three times more likely to develop occupational respiratory symptoms [51]. It is also supported by study done in Ethiopia [23]. This could be due to the fact that workers who had previous exposure tend to believe that they adapt to the dust or other hazards and ignore the use of personal protective equipment, which enhance the development of occupational respiratory symptoms [23].

## Strength and limitation of the study

This systematic review and meta-analysis rigorously followed PRISMA guideline at all steps. However, all the articles included in this systematic review and meta-analysis were cross-sectional, which limits the causality of predictors on occupational respiratory symptoms. Even though the scope of the study is country-wide, it does not include all regions due to the absence of studies in many regions of the country.

## Conclusions

High prevalence of occupational respiratory symptoms was recorded among industry workers in Ethiopia. Absence of personal protective equipment, lack of occupational health and safety training, previous dust exposure, poor working environment, having more than five years of work experience, being smoker, and previous respiratory diseases were factors associated with occupational respiratory symptoms. The provision of personal protective equipment and improvement of working environments by the government, industry owners, ministry of health, and other stakeholders are crucial in reducing occupational respiratory symptoms. Additionally, prioritizing occupational health and safety training for industry workers can help prevent and mitigate the impact of occupational respiratory diseases.

## Supporting information

S1 Table PRISMA-P 2009 checklist. (DOC)

S2 Table Results of JBI quality assessment. (DOCX)
