# Supplementary material for: Prevalence of occupational respiratory symptoms and associated factors among industry workers in Ethiopia: A systematic review and meta-analysis
Source: PLoS One. 2023 Jul 13;18(7):e0288238. doi: 10.1371/journal.pone.0288238 (PMC10343155; doi:10.1371/journal.pone.0288238)
Supplement: S2 Table — (DOCX) [file pone.0288238.s002.docx]

**Results of JBI Quality Assessment**

| Studies | Clear eligibility criteria | Description of study subject and study setting | Valid and reliable method to measure the exposure | Standard criteria used for measurement of the condition | Identification of confounding factors | Develop of strategies to deal with confounding factors | Valid and reliable method to measured outcomes | Appropriate statistical analysis | Total score out of 8 | Level of bias |
| --- | --- | --- | --- | --- | --- | --- | --- | --- | --- | --- |
| Alemu *et al* | Yes | Yes | Yes | No | N/A | No | Yes | Yes | 6 | 75% |
| Derso *et a*l | Yes | Yes | Yes | Yes | N/A | Yes | No | Yes | 7 | 87.5% |
| Abaya *et al* | Yes | Yes | No | Yes | N/A | No | Yes | Yes | 6 | 75% |
| kifle *et al* | Yes | Yes | Yes | Yes | N/A | Yes | Yes | Yes | 8 | 100% |
| Soyum *et al* | No | Yes | Yes | Yes | N/A | Yes | Yes | Yes | 7 | 87.5% |
| Alemseged *et al* | Yes | Yes | Yes | Yes | N/A | Yes | Yes | Yes | 8 | 100% |
| Awoke *et al* | Yes | Yes | Yes | No | N/A | Yes | Yes | Yes | 7 | 87.5% |
| Wami *et al* | Yes | Yes | Yes | Yes | N/A | No | Yes | Yes | 7 | 87.5% |
| Gizaw *et* al | Yes | Yes | Yes | Yes | N/A | Yes | No | Yes | 7 | 87.5% |
| Dalju et al | Yes | Yes | Yes | Yes | N/A | Yes | Yes | Yes | 8 | 100% |
| Jabur et al | Yes | Yes | No | Yes | N/A | Yes | Yes | Yes | 7 | 87.5% |
| Lagiso et al | Yes | Yes | Yes | Yes | N/A | Yes | Yes | Yes | 8 | 100% |
| Mekonnen et al | Yes | No | Yes | Yes | N/A | Yes | Yes | Yes | 7 | 87.5% |
| Tefera et al | Yes | Yes | No | Yes | N/A | No | Yes | Yes | 6 | 75% |
| Mekasha et al | Yes | Yes | Yes | Yes | N/A | No | Yes | Yes | 7 | 87.5% |
